# A Novel 3D Fibril Force Assay Implicates Src in Tumor Cell Force Generation in Collagen Networks

**DOI:** 10.1371/journal.pone.0058138

**Published:** 2013-03-11

**Authors:** Robert J. Polackwich, Daniel Koch, Richard Arevalo, Anne M. Miermont, Kathleen J. Jee, John Lazar, Jeffrey Urbach, Susette C. Mueller, Ryan G. McAllister

**Affiliations:** 1 Physics Department, Georgetown University, Washington, DC, United States of America; 2 Oncology Department, Lombardi Comprehensive Cancer Center, Georgetown University Medical Center, Washington, DC, United States of America; 3 Surgery Branch, National Cancer Institute, National Institutes of Health, Bethesda, Maryland, United States of America; University of Bergen, Norway

## Abstract

New insight into the biomechanics of cancer cell motility in 3D extracellular matrix (ECM) environments would significantly enhance our understanding of aggressive cancers and help identify new targets for intervention. While several methods for measuring the forces involved in cell-matrix interactions have been developed, previous to this study none have been able to measure forces in a fibrillar environment. We have developed a novel assay for simultaneously measuring cell mechanotransduction and motility in 3D fibrillar environments. The assay consists of a controlled-density fibrillar collagen gel atop a controlled-stiffness polyacrylamide (PAA) surface. Forces generated by living cells and their migration in the 3D collagen gel were measured with the 3D motion of tracer beads within the PAA layer. Here, this 3D fibril force assay is used to study the role of the invasion-associated protein kinase Src in mechanotransduction and motility. Src expression and activation are linked with proliferation, invasion, and metastasis, and have been shown to be required in 2D for invadopodia membranes to direct and mediate invasion. Breast cancer cell line MDA-MD-231 was stably transfected with GFP-tagged constitutively active Src or wild-type Src. In 3D fibrillar collagen matrices we found that, relative to wild-type Src, constitutively active Src: 1) increased the strength of cell-induced forces on the ECM, 2) did not significantly change migration speed, and 3) increased both the duration and the length, but not the number, of long membrane protrusions. Taken together, these results support the hypothesis that Src controls invasion by controlling the ability of the cell to form long lasting cellular protrusions to enable penetration through tissue barriers, in addition to its role in promoting invadopodia matrix-degrading activity.

## Introduction

Tumor cells exert measurable forces on their environment, both for cell movement and to reshape the surrounding matrix. Specific mutations may alter the force generation and mechanotransduction mechanisms in tumor cells, with significant implications for invasiveness. While previous assays allow for the study of cell-ECM interactions, these studies do not involve fibrillar environments.

The mechanical interaction between cells and their environment is usually mediated by the cellular force generation machinery, which exerts traction forces on the surrounding environment [1], [2]. Initial studies of traction forces monitored the wrinkling of thin, flexible sheets [3]. The introduction of uniform elastic films of controllable stiffness combined with advances in microscopy and analysis approaches have enabled researchers to map traction forces on flat surfaces with high spatial and temporal resolution [2], [4], [5], and even to map the forces propagating from cells on the 2D surface down into the surface [6]. However, these approaches all place the cells on a two-dimensional environment. Another approach, embedding cells in a three-dimensional (3D) hydrogel with known mechanical properties [7], allows for the measurement of cell force generation in a 3D, non-fibrillar environment.

However, many dynamic cellular processes, including invasive steps of metastasis, occur within a fibrillar environment of biopolymer proteins, such as collagen. The approach described here represents the first assay that measures the forces exerted by cells within a 3D fibrillar environment.

The tyrosine kinase Src has long been linked with proliferation, invasion, and metastasis [8]–[10] of cancer cells and is now considered an important drug target in solid tumors such as breast cancer [11]–[14]. Here, we manipulated the activity of Src within MDA-MB-231 tumor cells by transfecting fluorescent wild-type and constitutively active Src, and we observed and measured, using our novel assay, the effect on the force exertion and migration of the cells through a 3D fibrillar collagen network.

Src family kinases are intimately involved in cell migration that is regulated downstream of integrins. Src is required for receptor-like tyrosine phosphatase alpha (RPTPα)signaling during force production at cell contacts, and Src participates in signal transduction pathways intersecting other tension-sensing molecules such as talin and p130CAS during cellular response to matrix tension [15]–[19]. The formation of focal complexes during the early stages of the migration process are initiated by Src activity; Src family members are also involved with focal complex maturation to focal adhesions, focal adhesion turnover, and consequently force and rigidity response [20]–[23]. Src is required for invasive processes including protein degradation during cell migration through dense extracellular matrix (ECM), a process comprising invadopodia (cellular membrane protrusions that facilitate invasion) initiation, maturation and acquisition of degradation activity [24]–[26]. Localization of MT1-MMP (MMP-14), an integral membrane metalloproteinase that is required for invadopodia formation, requires Src [24].

Currently, an enhanced understanding of these events has led to considerable interest in the study of mechanotransduction signaling pathways and clinical applications [19], [27], [28]. Src itself is activated by force exerted through cellular adhesion to the matrix and is required for actin polymerization following integrin clustering and activation [29]–[31]. In addition to cell surface activation of Src [29], Na and colleagues provide evidence that myosin II and “tensed” actin cytoskeleton are required for rapid, localized Src activation in the cytoplasm by mechanical force [30]. A tensionally-integrated cytoskeleton (described by “tensegrity” [32]) indeed is critical to cellular response to forces, and to cell migration and invasion within a 3D ECM. Actin polymerization is required to form filopodia, invadopodia, and pseudopodial processes for the protrusion of membranes during cell migration and invasion [33]. Phosphorylation of cortactin by Src is critical for actin polymerization during the maturation of invadopodia to degradation-competence [34]. Myosin is also a prominent player in mechanotransduction, participating in substrate rigidity feedback, lamellipodial contraction, and force-induced or mechanical adaptation at focal adhesions and within cells [22], . Myosin II isoform switching mediates invasiveness of breast cancer cells during epithelial-to-mesenchymal transition EMT induced by TGF-beta [40].

Observed on 2D gelatin (denatured collagen) surfaces, MDA-MB-231 breast cancer cells exogenously expressing either forms of Src, wild type or constitutively active, have elevated levels of cell surface matrix-degrading proteases such as MT1-MMP and form more invadopodia than the parental cell line [24]. Manipulation of myosin IIB expression affected migration in 3D models but not in two dimensions, revealing the potential deficiencies of our understanding of epithelial cell invasion derived from 2D models [40]. The important implication of the results of this and similar studies is that cancer invasion must be modeled using 3D matrices designed to mimic the *in situ* microenvironments of invading tumor cells. The parameters of invasion must be carefully isolated, and ultimately their relevance to clinically significant cancer cell invasion understood. Although substrate rigidity and force define form through tyrosine phosphatase and kinase pathways and have primarily been studied in 2D, modeling and defining the differences in migration and invasion in 3D environments is critical [23], [28], [41], [42].

## Results

### Characterization of Breast Cancer Cell Clones Stably Expressing Src

**Figure 1:**
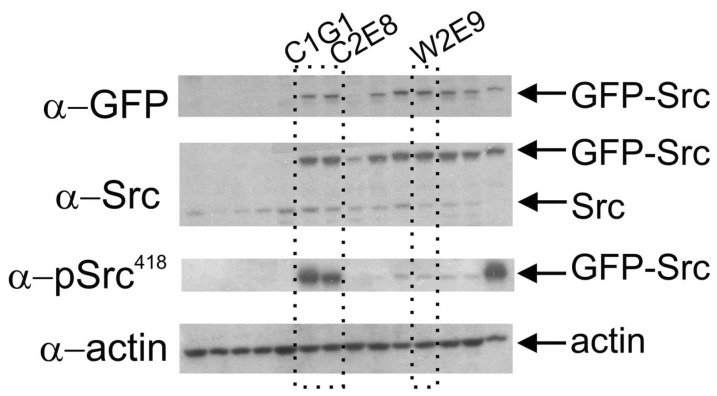
Protein levels of Src in GFP-Src transfectants. Lysates from clones of GFP-Src transfectants (30 µg each) were compared by immunoblotting using anti-GFP (α-GFP), anti-Src (α-Src), anti-pSrc^418^ (α-pSrc^418^), and anti-actin (α-actin). Three clones were chosen for study (C1G1 and C2E8 expressing GFP-ca-Src and W2E9 expressing GFP-wt-Src).

To study the role of the Src kinase protein on mechanotransduction and motility, we generated stable MDA-MB-231 breast cancer cell clones with similar expression of GFP-tagged wild type Src protein (GFP-wt-Src, W2E9 clone) and a GFP-tagged mutant of Src protein, c-Src(Y527F), rendering it constitutively active (GFP-ca-Src, C1G1 and C2E8 clones) (Fig. 1, α-Src and α-GFP). Moreover, GFP-ca-Src clones expressed constitutively active Src protein and demonstrated a higher level of active Src as compared to wt-Src as expected (Fig. 1, α-pSrc^418^).

### Localization of GFP-ca-Src in Protrusions, Focal Adhesions, and Invadopodia

**Figure 2:**
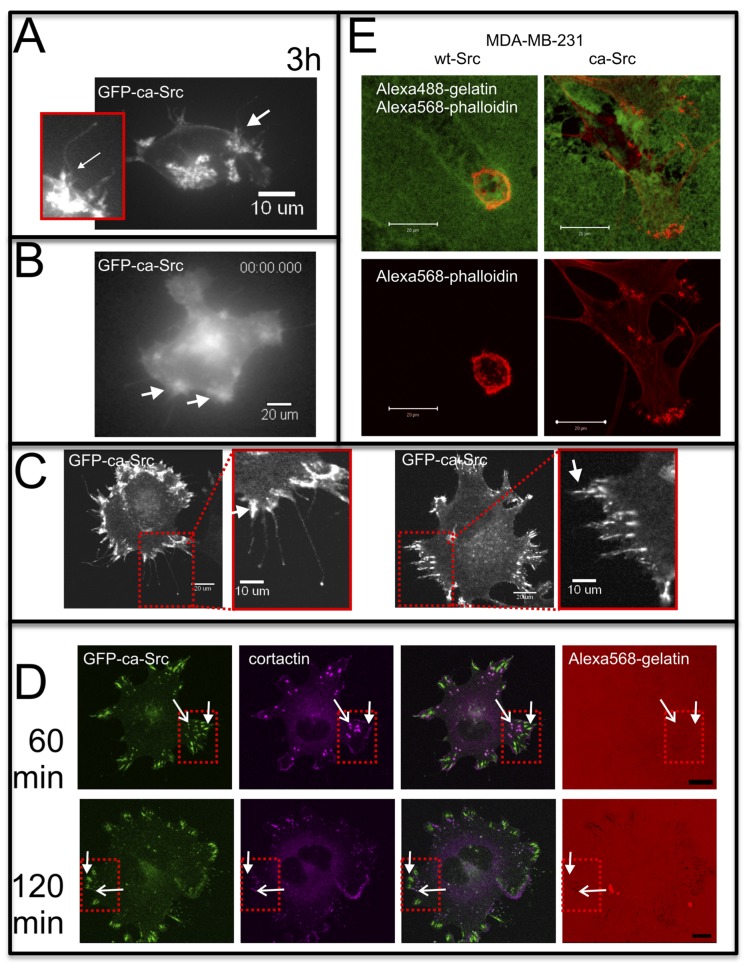
Localization of GFP-ca-Src at the plasma membrane and in focal adhesions and invadopodia core complexes associated with matrix degradation. MDA-MB-231 cells expressing GFP-ca-Src (A-D) or ca-Src (E) were cultured on glass (B), 2D collagen monomer layer (A, C), or crosslinked gelatin (E, D) and imaged using a customized Perkin Elmer spinning disk (A, C), laser scanning confocal (E, D), or epifluorescence widefield (B) microscope. Arrows in (C) indicate sites of concentrated GFP-ca-Src localization. Open arrows in (D) indicate invadopodia core complexes and closed arrows focal adhesions. (A and C) were cultured for about 2 h, (B and E) were cultured overnight, and the cells in (D) were cultured for 60 and 120 min, as indicated. Scale bar = 20 µm (C, B, E) or 10 µm (D, C insets). See also Movies S1, S2, and S3.

**Figure 3:**
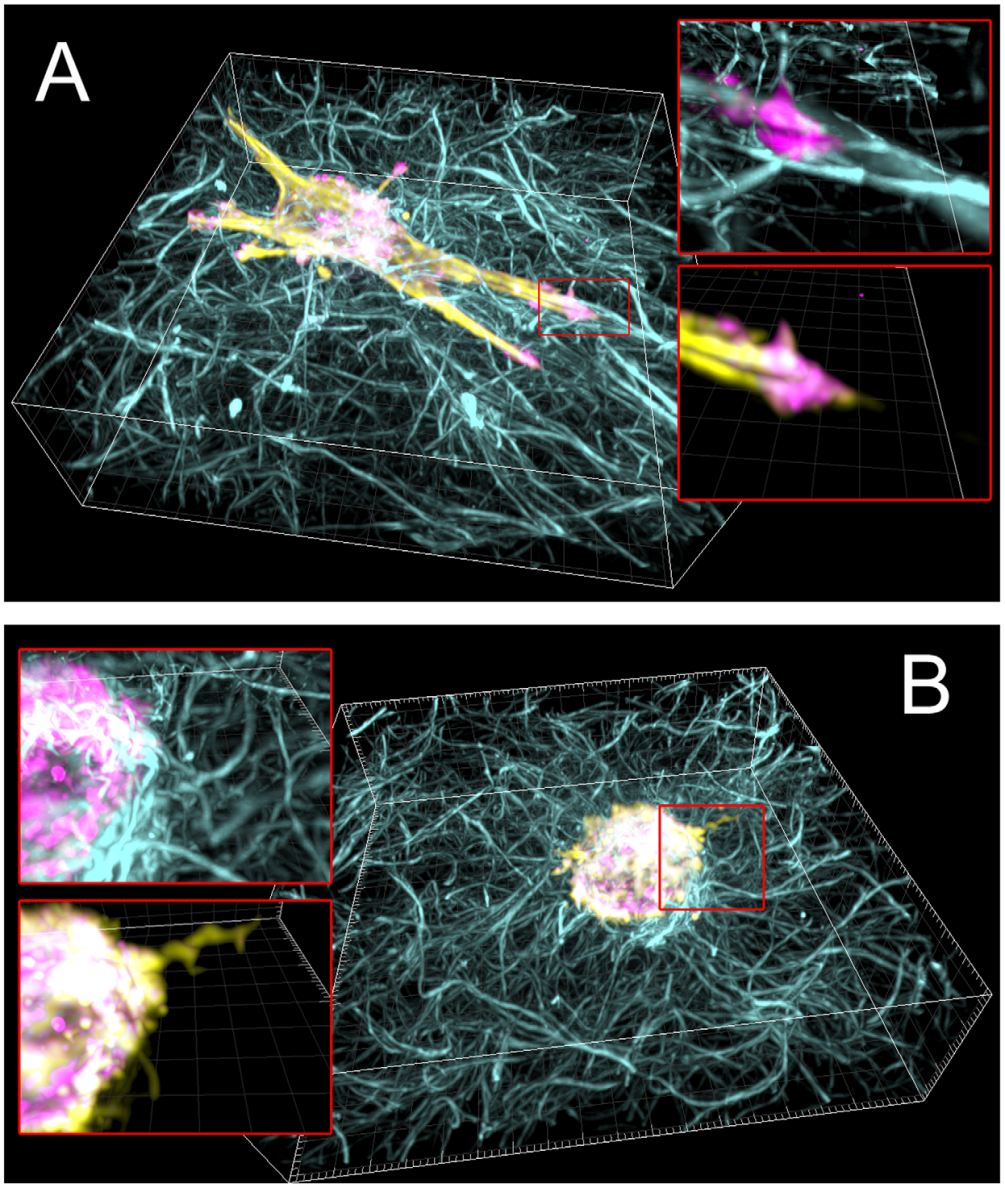
3D views of example fixed cells. Examples of each cell line fixed for the Src localization analysis (Fig. 8). **Cyan**: collagen fibrils. **Yellow**: Actin labled by Alexa-Fluor 568 Phalloidin. **Magenta**: eGFP-Src, (A) eGFP-ca-Src, (B) eGFP-wt-Src. In both cell types, F-actin is localized primarily at the cell periphery. Insets show zoomed in regions two-colors at a time to illustrate typical locations of Src and F-actin in protrusions and relative alignment of protrusions to collagen. See Fig. S2 for gallery views of individual z-slices, and Movies S8 and S9 for rotating 3D views. Cells were fixed after 6 hours in the 3D collagen network. Use of a higher-N.A. objective in the live-cell allows for better resolution of both Src position (see methods).

To characterize the behavior of the GFP-tagged wt-Src and ca-Src, we performed experiments to explore their localization in cells in 2D and 3D settings. MDA-MB-231/GFP-ca-Src cells were cultured under a variety of conditions to obtain high-resolution images of cellular protrusions. GFP-ca-Src was localized mostly at the cell surface and was clustered at sites associated with fine protrusions within 3 hours of the time when cells were cultured within a sandwich of collagen (maximum intensity projection of a z-stack of a living cell, Fig. 2, A). Cells cultured in the 3D collagen networks for 6 hours and then fixed also showed GFP-ca-Src localized mostly in membrane protrusions contrasted with GFP-wt-Src, which was localized mostly intracellularly (Fig. 3; Fig. S2, Movies S8 and S9). Time lapse epifluorescence imaging of GFP-ca-Src cells after overnight culture on glass revealed the dynamics of the fine, filopodia-like protrusions at sites of active membrane ruffling as well as an intracellular vesicular pool of GFP-ca-Src (Fig. 2, B and Movie S1). Some cells also contained GFP-ca-Src localized in focal adhesions and invadopodia core complexes (terminology of [43]) adjacent to dynamic protrusions (Movie S2). Confocal spinning disk imaging of these cells at longer time points revealed clustering of GFP-ca-Src at cell margins associated with filopodia-like extensions (Fig. 2, C left panels, arrow) and focal adhesion-like protrusions (Fig. 2, C, right panels, arrow). As previously demonstrated, cortactin identified sites of fluorescent crosslinked gelatin matrix degradation by invadopodia that become conspicuous by 90–120 min of culture on these crosslinked matrices (Fig. 2, D) [24], [44]. The visibility of the localization of fluorescent GFP-ca-Src after transfection clearly implies an association with both invadopodial core complexes (Fig. 2, D, open arrows) and focal adhesions (Fig. 2, D, closed arrows). On a thicker version of the 2D, glutaraldehyde-crosslinked, fluorescent gelatin matrix, the tracks left behind by migrating proteolytic MDA-MB-231/GFP-ca-Src or GFP-wt-Src cells stained for F-actin revealed cell size differences between the two that were also observed in 3D fibrillar collagen (Figs. 2 E and 3). In 3D fibrillar collagen culture, the GFP-ca-Src cells were larger (both soma and extensions) than the GFP-wt-Src cells (Fig. 3). Likewise, on the 2D matrix described above, the GFP-wt-Src cells were smaller and often left shallow tracks, whereas the GFP-ca-Src cells were larger and excavated larger holes (Fig. 2, E). Staining of F-actin using Alexa Fluor 568-phalloidin revealed the cell surface focused cytoskeleton associated with protrusions and matrix degradation (Fig. 2, E). In summary, MDA-MB-231/GFP-ca-Src and MDA-MB-231/GFP-wt-Src cells had mobile cell surface protrusions that were linked with matrix degradation (Fig. 2, and see [43], [45]).

### Measurement of Cell Force Generation in 3D Fibrillar Collagen

To focus on the role of Src tyrosine kinase in tumor cell invasion during migration through the stromal environment, we utilized a collagen type I matrix affixed to a relatively compliant polyacrylamide (PAA) gel in which fluorescent tracer beads were embedded. The collagen network was constituted from acid-extracted collagen, and thus requires MT1-MMP for invasion [46]. By tracking the x, y, and z motion of the beads in the PAA gel, we were able to measure the 3 dimensional stress caused by the tension generated by cells located a few microns above the PAA in the 3D collagen gel. To determine the specific contribution of the Src signaling pathway in cell force-generation in 3D collagen, we examined migration velocity and directionality, length and duration of membrane protrusions, and strength of force generation during migration for breast cancer cells transfected with either wild type GFP/c-Src or constitutively activated GFP-c-Src(Y527F).

**Figure 4:**
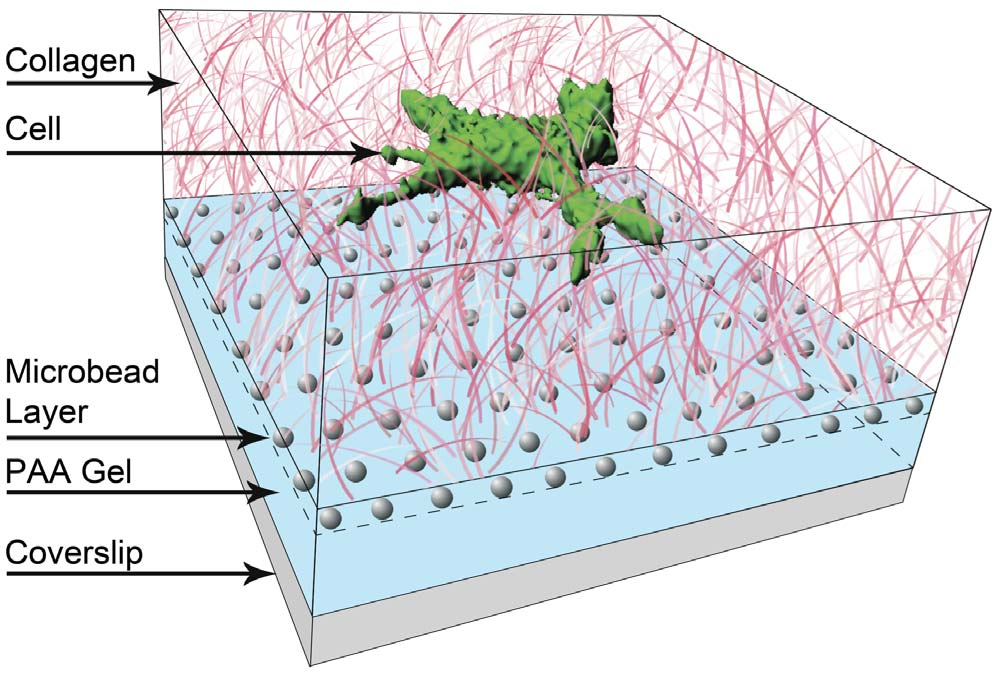
A cartoon depiction of the assay. The bottom layer (blue) is a soft (Young’s modulus ∼ 100 Pa) polyacrylamide (PAA) gel seeded with fluorescent tracer beads (gray). Above that (pink) is an approximately 80 µm thick network of fibrilized collagen I (2 mg/mL). Each imaged data series represents a 3D+time, 3 fluorescence channel movie of a single cell (green), the collagen fibrils, and the motions of the beads. Cell nuclei are typically about 5 µm above the PAA gel.

Fig. 4 shows a cartoon of the 3D fibril force assay used in these experiments. The collagen is fluorescently labeled such that the fibrils may be observed and their displacements measured. The PAA gel is seeded with fluorescent tracer beads (grey) that allow for the displacements of the gel to be measured with sub-pixel resolution. See the Materials and Methods sections for more details; a detailed protocol is included in the Supporting Information.

**Figure 5:**
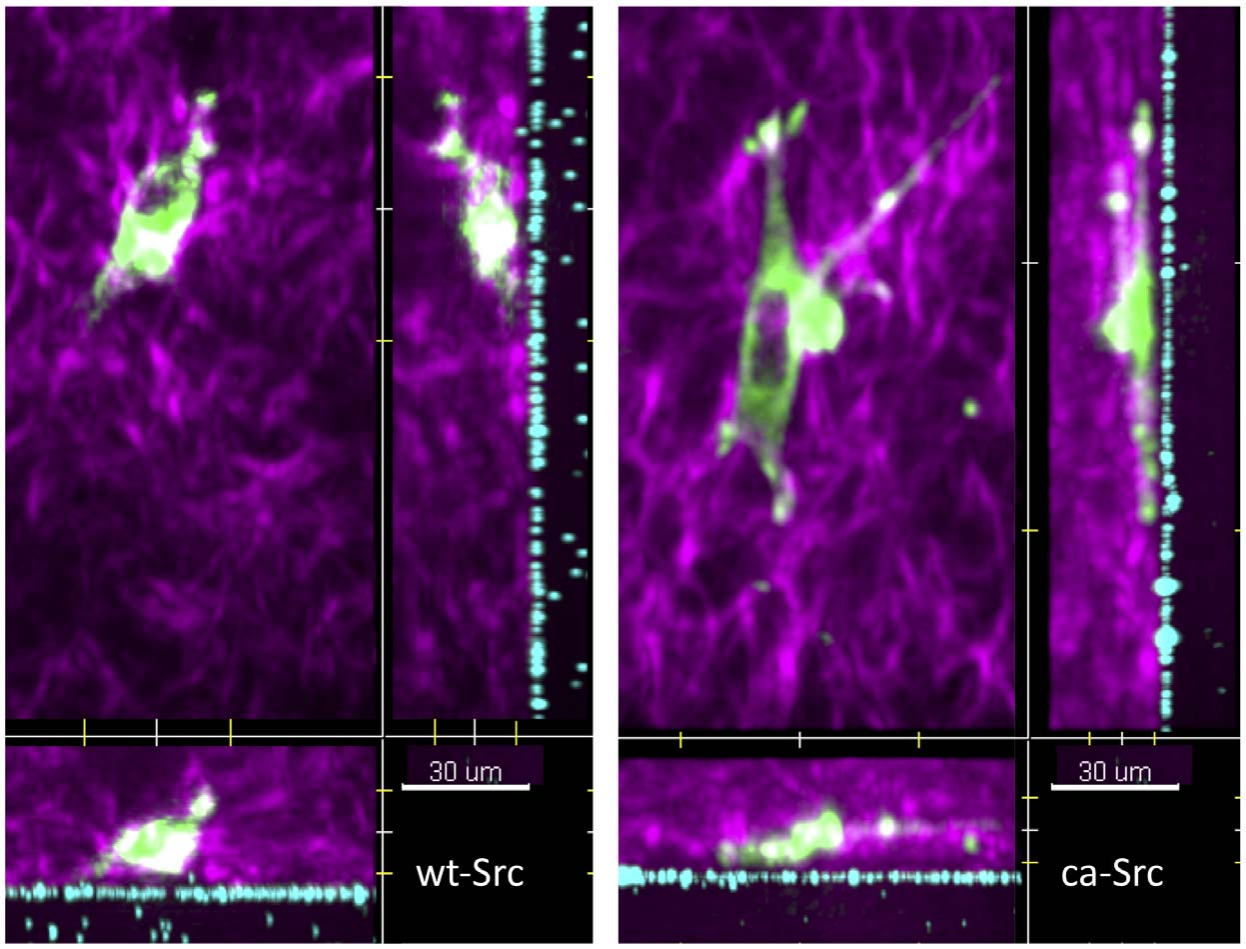
XY, XZ, and YZ maximum intensity projections of cells in the assay. LEFT: Three maximum intensity projections views of an MDA-MB-231 cell stably expressing GFP wild-type Src (green) in fibrillar type 1 rat tail collagen (magenta) on an approximately 50 µm thick PAA gel embedded w/fluorescently labeled 0.5 µm diameter microbeads (blue). RIGHT: The same projections of a MDA-MB-231 cell stably expressing GFP constitutively active Src (green). The matrix is submerged in media, maintained at 37 deg C, in an atmosphere of 5% CO_2_. See Movies S4 and S5 for 3D movies of the full time series.

Fig. 5 shows maximum-intensity projections (xy, xz, and yz) of 3D images of both GFP-wt-Src and GFP-ca-Src cell types in the 3D fibril force assay. Movement and membrane extensions of the cell (green) were observed simultaneously with the collagen network (magenta) and motions of the tracer beads (cyan) in the PAA gel.

**Figure 6:**
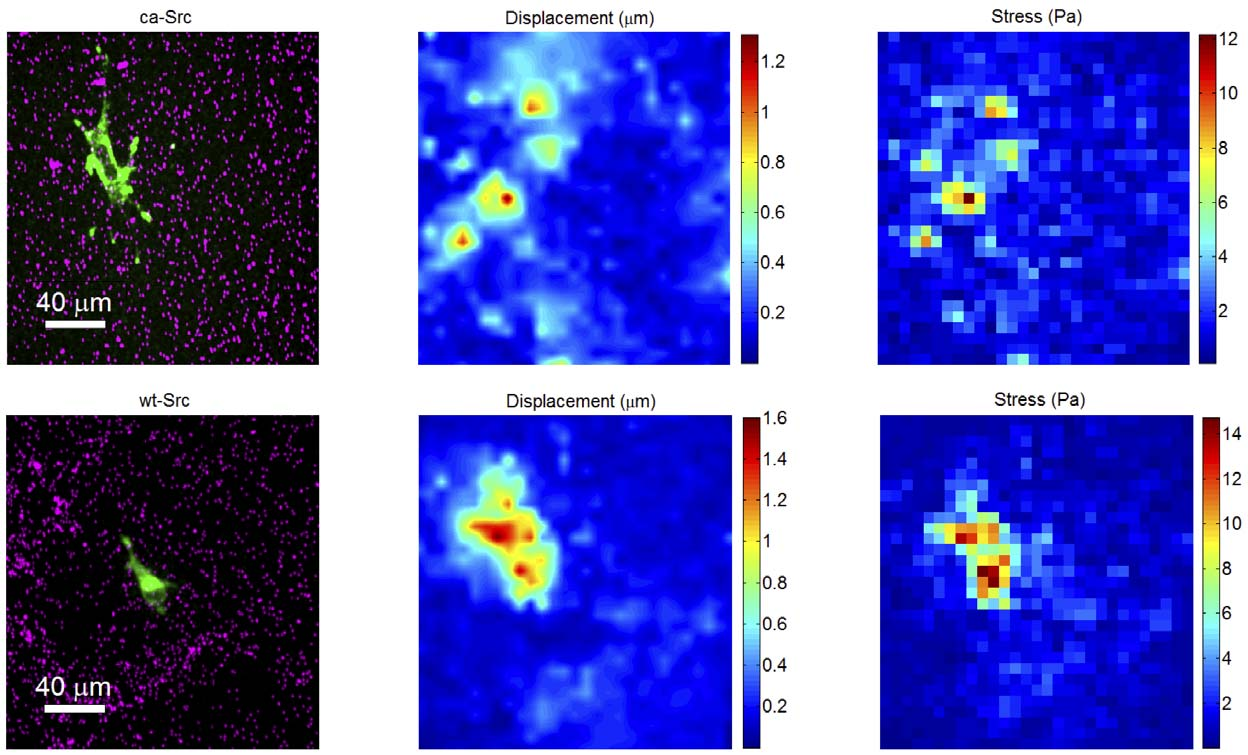
Graphical depiction of the analysis of cell-induced forces. Note that the analysis is performed in three dimensions with 3D data, even though the depiction here appears two-dimensional. **Top Row**: an MDA-MD-231 cell stably expressing GFP-constitutively active Src (GFP-ca-Src). **Bottom Row**: an MDA-MD-231 cell stably expressing GFP-Src (GFP-wt-Src). **Left Column**: 2D max intensity projection. *Green*: fluorescent image of GFP-constitutively active Src in an MDA-MD-231 cell; *Cyan*: beads in the PAA gel beneath the cell. The collagen channel has been suppressed for clarity. **Middle Column**: Heat map of the reconstructed amplitude of 3D deformation of the gel due to forces exerted by the cell, as calculated from the gel stiffness and the changes in bead positions at this time-point relative to the reference time-point. **Right Column**: Heat map of the amplitude of the 3D stress field at the gel surface caused by forces propagated from the cell by fibrils of the collagen gel, calculated from the displacement field (middle). See Movies S6 and S7 for movies showing the analysis of the full time series.

Fig. 6 illustrates the process of calculating the stress (force/area) acting at the collagen-PAA interface. The cells exerted forces on the ECM that were transmitted through the collagen fibrils to exert stress on the PAA gel. In brief, the 3D displacement of each bead was measured in each image to determine the deformation field of the PAA gel surface in three dimensions (both lateral and vertical displacements are measured). Knowing the induced deformation field and the mechanical properties of the PAA gel, we recovered the corresponding 3D components of the stress field (see Materials and Methods). Inhomogeneity of the bead distribution in the PAA gel (as seen in the bottom left panel) was due to the random locations of the beads during gelling and did not typically interfere with particle tracking; we also measured the stiffness of PAA gels with varying bead densities (from visually sparser than found in our experiments to visually denser than found in our experiments) using a rheometer, and found no discernible difference in the Young’s modulus (see Fig. S1).

### Constitutively Active Src Results in the Generation of Stronger Forces than Wild-type Src, but does not Significantly Affect Migration Speed

**Figure 7:**
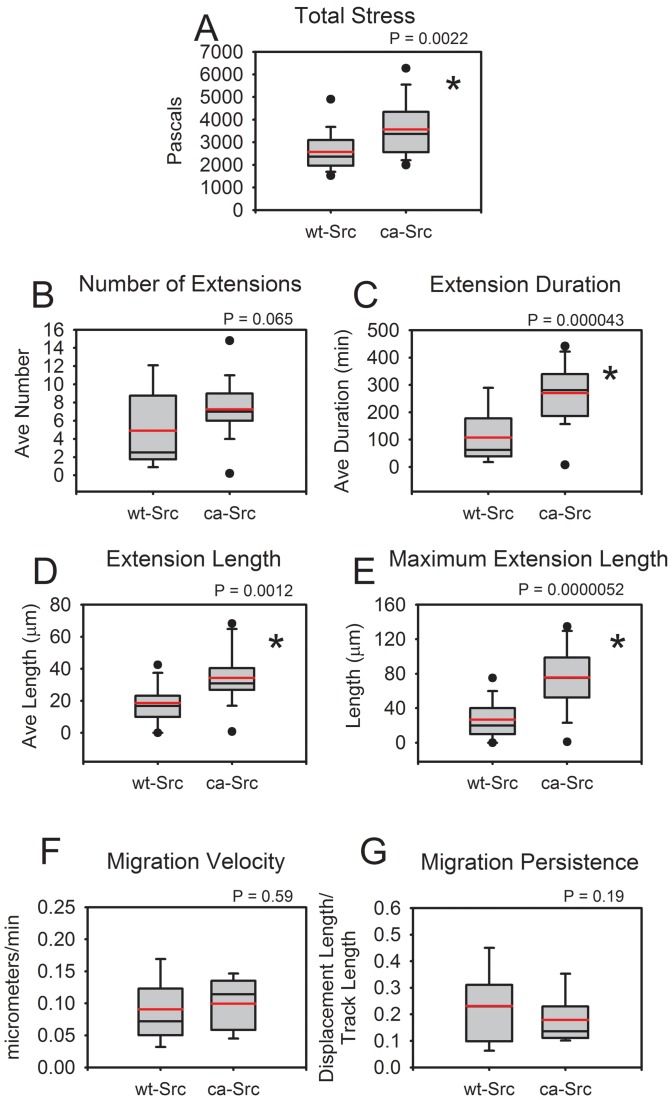
Assay results. MDA-MB-231 cells transfected with GFP-tagged constitutively active Src exert nearly twice as much total force on the gels via the collagen network as MDA-MB-231s transfected with GFP-tagged wild-type Src (A, p<0.005), while both cell populations travelled at similar speed (F) and with similar migration persistence (G). Compared with the same cell type transfected with GFP-tagged wild-type Src, MDA-MB-231 cells transfected with GFP-tagged constitutively active Src produce extensions that are longer in space (D, p<0.01 E, p<0.00001), and last longer in time (C, p<0.00005) while the number of cells studied was not sufficient to show a difference in overall number of extensions (B). Total stress at each time point was calculated as the sum of the magnitudes of each 3D stress vector calculated on the gel surface, and then averaged over time for the graph. Migration persistence was calculated as total displacement divided by path arclength of the each cell track. Extensions were considered to be cell membrane protrusions that visible in fluorescence that extended for at least 10 µm beyond the presumed cell body, and were measured manually to within the nearest 5 µm. The heavy line is mean, the thinner line median, the box represents the standard deviation, bars represent the full range except for outliers, dots represent outliers. Twenty-one GFP-wt-Src cells and Twenty-four GFP-ca-Src cells were analyzed.

The time-average of the total stress magnitude exerted by each cell population is shown in Fig. 7A. Total stress magnitude was calculated as the sum of the magnitudes of each 3D stress vector calculated on the gel surface, averaged over time. Transfection with constitutively active GFP-ca-Src nearly doubled the total stress exerted by the MDA-MB-231 cells relative to cells transfected with wild-type GFP-wt-Src (p<0.005); total stress for GFP-wt-Src cells was 2574.7 kPa (S.E. 182.1) but for GFP-ca-Src cells was 3569.3 kPa (S.E. 237.3). However, as shown in Fig. 7F, the mean velocity of both cell types is not statistically different (p>0.5); mean velocity for GFP-wt-Src cells was 0.09 µm/min (S.E. 0.01) and for GFP-ca-Src cells was 0.10 µm/min (S.E. 0.01). While average migration persistence was lower in ca-Src cells than wt-Src cells, the result in this study was not statistically significant (0.23, S.E. 0.03 for wt-Src versus 0.18, S.E. 0.02 for ca-Src, p>0.1).

### Constitutively Active Src Results in the Generation of Spatially Longer Membrane Extensions that also Last Longer than Wild-type Src, but does not Significantly Affect the Number of Protrusions Generated

Number, duration, and length of extensions are presented in Fig. 7 B, C, and D-E respectively. While MDA-MB-231 cells transfected with GFP-ca-Src and GFP-wt-Src failed to show a statistically significant difference (p>0.05) in number of extensions generated (4.89, S.E. 1.05) for GFP-wt-Src versus (7.25, S.E. 0.70) for GFP-ca-Src, the GFP-ca-Src cells generated extensions that were generally about twice as long in duration: p<5×10^−5^, 107.32 min (S.E. 25.06 min) for GFP-wt-Src versus 270.10 min (S.E. 24.33 min) for GFP-ca-Src. The length of the longest extensions of GFP-ca-Src cells was also about twice that of the longest extensions of GFP-wt-Src cells: p<1×10^−5^, 26.84 µm (S.E. 4.70 µm) for GFP-wt-Src versus 75.50 µm (S.E. 7.71 µm) for GFP-ca-Src. The average extension length of GFP-ca-Src cells was also twice that of GFP-wt-Src cells: p<0.002, 18.61 µm (S.E. 2.67 µm) for GFP-wt-Src versus 34.36 µm (S.E. 3.55 µm) for GFP-ca-Src. Extensions were considered to be cell membrane protrusions that were visible in fluorescence that extended for at least 10 µm beyond the presumed main cell-body. Using the measurement tools of Imaris (Bitplane), the length of a protrusion in 3D was manually determined from the pinch off area at the base of the main cell body to the tip to within the nearest 5 µm.

### Transfection with Constitutively-active Src was Located Preferentially in the Cell Membrane, Moderately Increased MT1-MMP Expression, and Resulted in More Polarized Cells than Transfection with Wild-type Src

**Figure 8:**
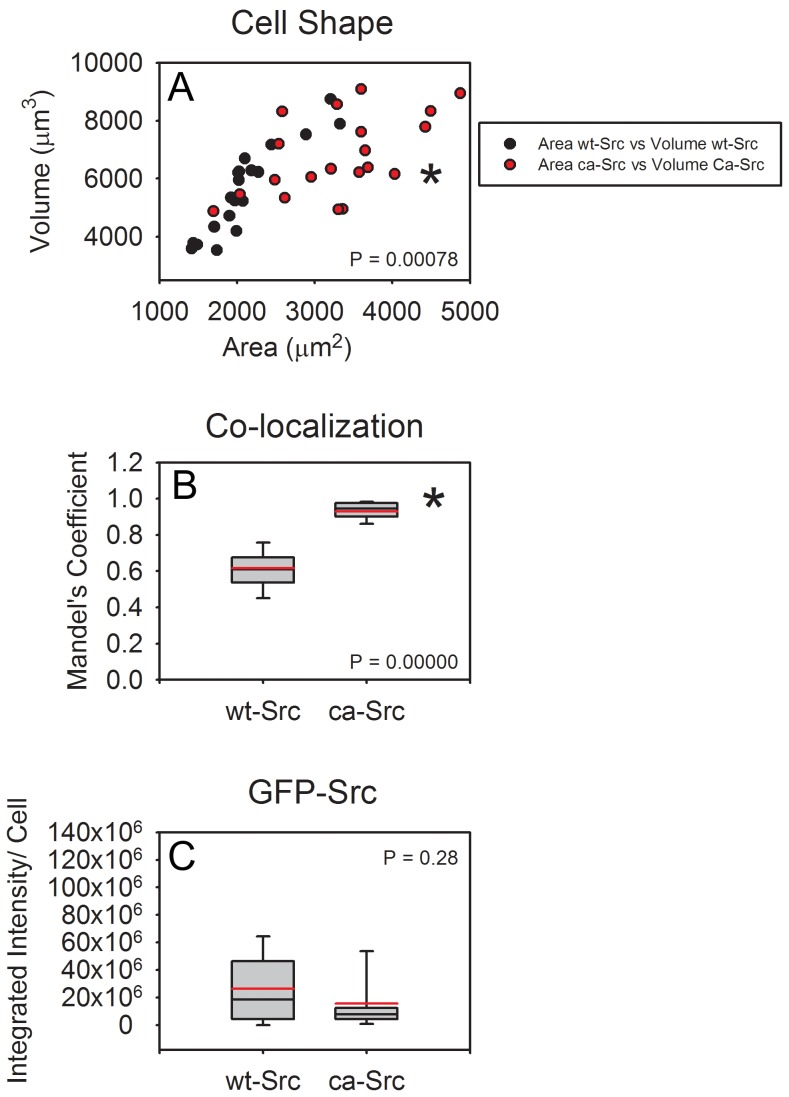
Cell Shape, Src location, and total Src. (A) MDA-MB-231 cells transfected with GFP-tagged constitutively active Src exert tended to have a higher surface area to volume ratio (p<.001) as well as tending to be larger in volume and have larger surface area,compared with MDA-MB-231s transfected with GFP-tagged wild-type Src. (B) Src was preferentially located in the membrane protrusions of cells (and was therefore colocalized with labeled actin with GFP-ca-Src whereas it was preferentially located in the cytosol of cells with GFP-wt-Src (p<.00001). (C) the difference in total GFP-Src in each cell type was not statistically different. Cells were fixed after 6 hours in the 3D collagen network; example cells are shown in Fig. 3.

Fig. 8A shows the surface area and volume of 19 (wt-Src) and 17 (ca-Src) cells fixed after 6 hours in the 3D fibrillar collagen. Cells with eGFP-ca-Src had higher surface area to volume ratios (p<0.001) and higher overall volumes than cells with eGFP-wt-Src. Fig. 8B reveals the co-localization of Src and F-actin, showing that ca-Src was significantly more localized to the cell membrane than wt-Src, which was mostly bound intracellularly (p<0.00001), though the average total amount of Src in each cell type (measured as fluorescent intensity) was not statistically differentiable between the two cell populations (p>0.2) (Fig. 8C). Fig. 3 shows 3D views of an example cell of each type. In both cell types, protrusions and collagen fibrils tended to co-align. Gallery views of individual z-slices can be viewed in Fig. S2, and 3D rotations can be viewed as Movies S8 and S9.

**Figure 9:**
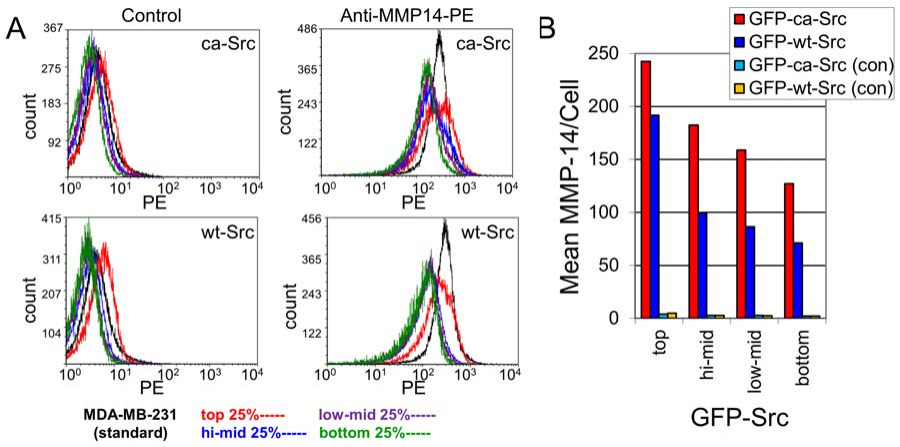
Cell surface MT1-MMP expression. (A) Flow cytometry was performed on GFP-ca-Src and GFP-wt-Src cells cultured to subconfluence in tissue culture flasks. The GFP-ca-Src and GFP-wt-Src expressing cells were harvested by trypsinization, washed, and then were labeled with anti-MMP-14/MT1-MMP-PE while cells were kept chilled on ice. Following immunostaining, the cells were washed, fixed and then subjected to FACS analysis gating cell fluorescence intensities into four groups: top, hi-mid, low-mid, and bottom 25 percentiles for GFP expression. Controls (at left; no antibody) were compared to anti-MMP-14/MT1-MMP-phycoerythrin (anti-MMP-14-PE, at right). (B) The mean fluorescence intensity per cell was plotted for anti-MMP-14-PE versus control (no antibody).

Fig. 9 shows FACS measurements of the amount of MT1-MMP in each cell type. While stable expression of eGFP-ca-Src did result in increased MT1-MMP expression relative to eGFP-wt-Src, the difference was less than 50%.

## Discussion

While previous assays allow for the study of cell-ECM interactions, those studies do not involve fibrillar environments. This study demonstrates the ability of the 3D fibrillar force assay to measure the forces exerted by tumor cells in fibrillar collagen. We utilized this assay to determine the effects of constitutively active Src (ca-Src) versus wild-type Src (wt-Src) expression. This assay successfully allows for the characterization of the forces generated on the ECM, as well as the measurement of the effect of molecular changes within the cell on the forces the cell exerts.

This study shows that, in a 3D fibrillar collagen network, stable expression of GFP-ca-Src substantially increased both the extension length and duration of extensions in MDA-MB-231 cells compared with the same cells stably expressing GFP-wt-Src. Surprisingly, the difference in Src activity, pull force, and extension length caused no significant change in cell motility. The main observed effect of the increased contractile force by the cells was greater deformation and rearrangement of the collagen fibrils.

In 2D cultures, increased ca-Src tyrosine kinase activity in MDA-MB-231 cells was associated with increased invadopodia formation and matrix degradation ([24], [44], [47] for review). In 3D, differences in migration rate were not apparent; GFP-wt-Src and GFP-ca-Src cells both migrated in 3D fibrillar collagen about 5–6 µm/h. During 3D invasion, migration might be significantly slower if proteolysis of the matrix and expression of MT1-MMP were the limiting factors for migration velocity. MDA-MB-231/ca-Src cells form more proteolytically active invadopodia compared with untransfected or wt-Src transfected cells in a 2D setting [24], [44] and we confirmed that they expressed a higher cell surface expression of MT1-MMP. Thus, it is plausible that no difference in migration rate was observed in 3D collagen between GFP-wt-Src and GFP-ca-Src cells because increased matrix proteolysis by GFP-ca-Src cells afforded a migration advantage even though GFP-wt-Src cells might have had a higher inherent translocation ability due to tightly regulated Src function. Published data supporting this possibility show that MT1-MMP is required for invadopodia-mediated matrix degradation [24], MT1-MMP is required for tumor cells to traverse collagen-rich ECM barriers [46], [48], [49], and invasion in collagen is sensitive to Src and protease inhibitors [50].

In the current study, we extend the understanding of the role of Src in 3D mesenchymal-like tumor cell invasion in collagen gels to include its function to regulate cell traction associated with cellular protrusions of longer duration and length. We found an average protrusion duration of about 270 min for GFP-ca-Src cells compared with 107 min for GFP-wt-Src cells and average protrusion maximum lengths of 76 µm for GFP-ca-Src cells compared with 27 µm for GFP-wt-Src cells. We further show that the cell protrusions contain focal adhesion-like structures and invadopodia core complexes based on GFP-Src colocalization with F-actin and that dynamic membrane ruffling and membrane extensions form associated with these adhesion sites. Traction forces and protrusion lengths and lifetimes were measured from images necessarily obtained with a wide field-of-view, circumstances under which invadopodia and matrix adhesions containing GFP-Src could not be resolved. We confirmed that GFP-ca-Src localizes to invadopodia and focal adhesions in 2D using higher resolution imaging of the same GFP-ca-Src cell line used in the traction force study. Similarly, cell protrusions in 2D and within 3D collagen contained clustered GFP-ca-Src near membrane protrusions that were highly dynamic. MDA-MB-231 cells and wt-Src transfectants form invadopodia in collagen 3D matrices where fine invadopodia membranes emanate from cortactin/actin/phosphoproteins-rich core structures [43], [45]. Schoumacher et al. found that invadopodia protrusions (stages 1 and 2) in invasive MDA-MB-231 and HCT116 cells were 1–7 µm long and 0.5 to 2 µm in diameter in 3D collagen culture [51]. The relationship of these thin protrusions to invadopodia was determined by the presence of MT1-MMP and cortactin as well as active c-Src [51]. c-Src, MT1-MMP and cortactin are well-documented components of invadopodia membranes [24], [44], [47], [52], [53]. MMP-dependent and degradation-associated long membrane extensions have long been observed in other 3D settings [25], [47]. We conclude that cell protrusions contain the potential for strong adhesions coupled with dynamic membrane movements that probably possess the matrix-degrading capabilities of invadopodia. The physical presence of Src at the membrane and at sites of adhesion and degradation in 3D collagen cultures together with data derived from 2D studies of cells support the interpretation that Src tyrosine kinase regulates the length and lifetime of protrusions in addition to its ability to regulate invadopodial proteolytic activity associated with those protrusions.

### What is the Source of the Enhanced Traction Produced in GFP-ca-Src Cells Compared with GFP-wt-Src Cells?

Since the number of protrusions was not significantly different between GFP-wt-Src and GFP-ca-Src cells, several possibilities exist to explain the role of Src in promoting cell force generation related to the observed changes in length and duration of cellular extensions. It is possible that longer protrusions may contain more adhesions and thus produce more force. GFP-ca-Src is localized in both focal adhesions and invadopodia core structures and thus probably influences their functions such as adhesion and degradation, respectively, during migration and invasion (this study and data not shown) [24], [44]. The fact that protrusions are of longer duration would also be expected to contribute to increased force production.

The participation of focal adhesions and their associated actin cytoskeleton in force generation is suggested by studies utilizing Rho-kinase (ROCK) inhibitors; in fibroblasts, traction forces on the extracellular matrix were associated with the extension of pseudopodial processes that could be inhibited using ROCK inhibitors [54], [55]. Although invadapodia are force-sensing structures which depend upon both Src and ECM mechanical properties, their ability to enhance force generation has not previously been studied [25], [56]–[59]. Thus the role, if any, of invadopodia in force generation in 3D is not clear and is further confused by the question of their expected involvement with protein degradation and its coordination with force production.

### What Regulates the Length of Protrusions?

Src family kinases are activated near the leading edge during cell protrusion; their activities correlate with velocity of lamellipodia extension and are inhibited by PP2, a Src inhibitor which slows cell protrusion [60]. In our study, the presence of constitutively active GFP-ca-Src, as opposed to regulated GFP-wt-Src, was sufficient to lengthen cell protrusions. Machesky and colleagues postulate that matrix remodeling is accomplished by “invadopodia equivalents” that might take the form of very small unobtrusive structures in some invasive tumor cells, but form more easily observed long membrane extensions in fascin-requiring invasion [61]. Thus, fascin, a PKC substrate, could act to stabilize actin to generate longer-lived or longer protrusions via crosstalk between Src and PKC [61]. Other mechanisms governed by Src that could influence protrusion lengths include effects upon substrates regulating actin polymerization and Rho family GTPases (see for example references contained in [25], [60]). Others have also described protrusions of difference lengths in MDA-MB-231 cells. Kikuchi et al. observed that long protrusions were associated with MDA-MB-231 cell invasion in 3D, whereas short protrusions could be formed by all the cell lines whether or not they were invasive, such as the non-invasive MCF- breast cancer cells; longer protrusions could be distinguished by their dependence upon tubulin and WAVE2 [62]. More recently, Schoumacher et al. demonstrated the requirement for filopodial actin machinery during invadopodia elongation. However, they demonstrated that further growth of invadopodia-associated membrane protrusions depended upon vimentin intermediate filaments and tubulin [51], [63]. Thus, there are multiple molecular mechanisms on which Src signaling may impinge to create longer, force-producing cellular protrusions.

### What are Potential Other Functions of Increased Traction Force and Membrane Protrusion Extensions?

Here we show that in 3D collagen matrices, Src stimulates increased traction force production. It is clear that Src plays a role in cell translocation through force production, but we have also seen that, when coupled to controlled matrix degradation, Src gives rise to invasion through the otherwise impenetrable ECM. Furthermore, Src-associated pull force and extension length may have effects on cell proliferation. Consistent with the role of Src in driving force production is that Src activation promotes cell surface expression of MT1-MMP in MDA-MB-231 cells on rigid 2D interfaces [24]. MT1-MMP is required for cell growth in 3D but not 2D environments [64]. Feedback to the cell which detects increased matrix rigidity activates cell proliferation signaling [65]. Coupled with likely higher levels of MT1-MMP at the cell surface and increased invadopodia formation, both cell growth and cell invasion would be elevated.

Alternatively, extensions and increased pull force might function to establish cell-cell interactions. Invadopodia in tumor cells and invasive podosomes in macrophages form long membrane extensions that extend from cell protrusions into and through the extracellular matrix [61], [66]–[68]. Although they are thought to function to degrade the matrix, these membrane extensions, together with the associated cytoskeleton (referred to as invadosomes), may have diverse functions in various cell types [25], [47], [69]–[73].

In conclusion we have demonstrated an entirely new approach to measuring cell-matrix interactions, allowing measurement of cell-induced forces in a 3D fibrillar environment. We have been able to quantify the role of Src activation in cell-fibril interactions for MDA-MB-231 tumor cells in a type I collagen matrix, and show that transfection with GFP-ca-Src doubles the average force generation in these cells compared to GFP-wt-Src.

## Materials and Methods

### Plasmids

pIGFP-N3-Src was a kind gift of John Woodward [1]. A point mutation was introduced into pIGFP-N3-cSrc by site-directed mutagenesis using the Quick Change mutagenesis kit according to the manufacturer’s instructions (Stratagene, La Jolla, CA, USA) as follows. A constitutively active pIGFP-N3-cSrc mutant (pIGFP-N3-cSrc-Y527F) was generated by site-directed mutagenesis using the following primer pair: 5′-CTCGACAGA GCCCCAGTTCCAGCCTGGAGAGAACC-3′ and 5′-GGTTCTCTCCAGGCTGGAACTGGG GCTCTGTCGAG-3′. Construct integrity was confirmed by DNA sequencing.

### Cell Lines

MDA-MB-231 c-Src transfectants were previously described [2], [3]. One GFP-wt-Src stable clone (W2E9) and two GFP-mutant ca-Src stable clones (C1G1 and C2E8) were generated from stable MDA-MD-231 breast cancer cell lines expressing GFP-wt-Src or GFP-ca-Src (mutant c-Src(Y527F)), respectively. Briefly, 300,000 MDA-MB-231 cells were transfected with 1 µg pIGFP-N3-cSrc or pIGFP-N3-cSrc-Y527F construct using 3 µL Fugene (Promega, Madison, WI, USA) following the manufacturer’s instructions, and selected with 500 µg/mL G418 (Invitrogen, Carlsbad, CA, USA). Subsequent enrichment for cells with similar expression levels of GFP-wt-Src or GFP-ca-Src was performed by fluorescence-activated cell sorting (FACS). Clones were generated by initial dilution and continuous selection in G418 and then characterized using western blotting. Cells from the human breast cancer cell line MDA-MB-231 or GFP-Src transfectants created using this cell line were thawed and maintained in modified IMEM (Invitrogen) supplemented with 10% fetal bovine serum and 1% L-glutamine.

### Western Blot

Cells were lysed in RIPA buffer (20 mM Na2HPO4, 250 mM NaCl, 1% Triton, 1% DOC, 0.1% SDS, protease inhibitor Complete Mini (Roche, Indianapolis, IN, USA), 10 mM sodium vanadate), sonicated, spun at 10,000 g for 10 min, and lysates were frozen at −80°C until future use. Protein concentrations were measured using DCTM Protein Assay kit (BioRad, Hercules, CA, USA). Lysates were boiled for 3 min and 30 µg of protein was run on an 8% Tris-Glycine gel (BioRad) and transferred to a PVDF membrane (Millipore, Billerica, MA, USA). The membrane was blocked with 3% BSA for 1 hr and probed with anti-Src M327 mAb (2.5 µg/mL, O/N 4°C, Calbiochem, Gibbstown, NJ, USA), anti-GFP (1∶2,000, O/N 4°C, Clontech, Mountain View, CA, USA), anti-pSrc (418) (1∶1,000, O/N 4°C, Biosource, Chevy Chase, MD, USA) and anti-actin I19 antibody (1∶500, 1 hr RT, Santacruz, Santacruz, CA, USA).

### Fluorescent Matrix Degradation Assay

Cells were cultured on thin, fluorescent, crosslinked gelatin matrices as previously described [24], [74]. Briefly, cells were seeded on the coverslips contained within a 12-well culture plate at a density of 70,000 cells per well. The cells were incubated at 37°C at 5% CO_2_ for the specified time. In one experiment (Fig. 2, E), cells were cultured on a thicker matrix [44], [75] in which the gelatin was applied in a thicker layer prior to fixation using 0.5% glutaraldehyde.

### Immunofluorescence Staining

Cells were fixed with 10% formalin (3.7% formaldehyde in PBS) for 10 min, permeabilized with 0.1% Triton in PBS for 5 min, and washed with PBS. The cells then underwent primary antibody incubation for 15 min with 1 µg/µL anti-cortactin clone 4F11 mouse monoclonal antibody (Upstate (Millipore, Billerica, MA)) diluted 1∶200 in 10% normal donkey serum in PBS. After a wash with PBS, the cells were labeled with a secondary donkey anti-mouse antibody conjugated to Cy5 and diluted 1∶200 for 15 min. Alternately, cells were stained for 20 min with Alexa Fluor 568-phalloidin (Molecular Probes®/Invitrogen, Life Technologies, Grand Island, NY). To facilitate observation through microscopy, the coverslips were mounted onto glass slides using Invitrogen Prolong Antifade Kit.

### Epifluorescence Widefield Time Lapse

Widefield epifluorescence (Fig. 2, B) was conducted using a 60×/1.4 N.A. objective on a Nikon TE300 inverted microscope equipped with an EM-CCD camera (Hamamatsu Photonics, Japan) and Lambda DG-4 illumination system (Sutter Instrument, Novato, CA) and run using Metamorph® Acquisition.

### Laser Scanning Confocal Imaging

Laser scanning confocal images of fixed and immunostained GFP-ca-Src cells (Fig. 2, D and E) were obtained using an Olympus Fluoview-FV300 laser scanning confocal with a 60×/1.4 N.A oil objective (Fig. 2, D) or a Zeiss LSM510 laser scanning confocal with a 63×/1.4 N.A. oil objective. Line scans were acquired sequentially (multitrack) using argon (488 nm), green helium-neon (543 nm), and red helium-neon lasers (633 nm).

### Spinning Disk Confocal Images of GFP-ca-Src Cells Cultured within a Collagen Sandwich

Collagen type I from rat tail (BD Biosciences) of concentration 4.27 mg/mL was kept at 4°C. A 2 mg/mL collagen type I solution (Collagen Solution A) was prepared and kept on ice under sterile conditions by mixing five reagents. The following were mixed in this order: 409 µL dH_2_O, 100 µL 10× Opti-MEM, 13 µL of a 7.5% NaHCO_3_ solution, 10 µL 100× antibiotic/antimycotic, and 468 µL of a 4.27 mg/mL collagen type I stock. A volume of 200 µL collagen solution A is needed per dish. Per each 14 mm glass coverslip-containing culture dish (MatTek Corporation, part no. P50G-0-14-F), 20 µL collagen solution A was spread evenly over each coverslip with pipette tip and placed in 37°C incubator for at least 10 minutes, and enough time for the collagen to completely polymerize. 4 mL of a ∼85,000 cells/mL suspension was pipetted into each dish, and all dishes were then placed in the 37°C incubator for 30 minutes to allow cells to sink into the collagen matrix. Afterwards, excess media was delicately aspirated and a final 180 µL layer of liquid collagen solution A was pipetted atop the original collagen layer, thereby sandwiching the cells between two layers of collagen type I. After a 15 min incubation at 37°C, 4 mL cell media was gently added to each dish to prevent desiccation of the matrix, and dishes were then placed in the incubator until imaged. For spinning disk imaging, a Nikon Eclipse TE2000-U was custom equipped with a Yokogawa CSU21 spinning disk with an iXon 887 back-thinned EM-CCD camera (Andor Technologies). GFP-ca-Src cells were imaged ∼2 hours after being seeded in Collagen Solution A (Fig. 2 A, C). All images were acquired with a 100x/1.49 N.A. oil objective.

### Polyacrylamide (PAA) Preparation and Plating

35mm glass bottom MatTek (MatTek, Ashland, MA) plates were wiped down with ethanol. After drying, the glass bottoms were functionalized with 2 mg/cm^2^ (5 mL of 1 mg/mL CellTak (BD Biosciences) in 200 mL diH2O) for 25 minutes and then washed three times with diH20. Eighteen mm glass top coverslips were wiped down with ethanol and then submerged in Sigmacote (Sigma-Aldrich) for 3 minutes to make the surfaces hydrophobic and then rinsed in dH_2_0 for 10 minutes. To make the polyacrylamide gel, 835 µL dH_2_0 was mixed in a centrifuge tube with 75 µL 40% Bio-Acrylamide and 70 µL 1% Bis-Acrylamide (Bio-Rad Laboratories, Richmond, CA). A volume of 15 µL FluoSphere microbead solution (0.5 mm, 540 nm; Invitrogen) was added to the mixture. The PAA mixture was desiccated for 15 minutes and then placed on ice for 5 minutes. The PAA crosslinking was initialized by the addition of 10 µL of 10% Ammonium Persulfate (10% w/v solution; Sigma-Aldrich) and 3 µL of *N,N,N,N*-tetramethylethylenediamine (TEMED; AcrosOrganics, Morris Plains, NJ). A volume of 6 µL PAA solution (for approximately a 50 µm thick gel) was pipetted onto the MatTek glass bottom coverslip and the top coverslip was placed onto the droplet with the hydrophobic side against the PAA gel. During PAA gellation, the MatTek plates were inverted to achieve a more uniform surface bead density and covered with a wet paper towel to provide a humid environment for polymerization. The bead density still varied somewhat across each sample, therefore to determine if the bead density affected the PAA stiffness, we measured the stiffness of PAA gels with varying bead densities (from visually sparser than found in our experiments to visually denser than found in our experiments), and found no discernible difference in the modulus (see Fig. S1). The plates were allowed to polymerize this way for approximately 15 minutes. After polymerization, 3 mL diH_2_0 was pipetted into the dishes and left for 30 minutes. While submerged, the top coverslips were removed carefully using tweezers. The plates could be stored overnight with the addition of 30 µL PSF (antibiotic-antimycotic formulation; penicillin, streptomycin, fungizone). The PAA gel surface was functionalized again with CellTak as described above to facilitate collagen binding to the PAA surface.

### Collagen, Cell Preparation and Plating

To make fluorescent collagen, 0.35 mL 10× PBS was mixed with 0.081 mL 1N NaOH in a tube before adding 3.5 mL acid extracted rat tail tendon collagen I stock (BD Biosciences Discover Labware, Bedford, MA) and vortexing. A volume of 1.5 mL was plated into two 35 mm Mat-Tek dish and gelled overnight in the dark at 21°C. Plates were washed with sterile 0.1M NaHCO_3_ 5 times for 30 minutes each, yielding a pH of approximately 8.3. A volume of 1 µM CF633 (Biotium) carboxylic acid, succinimidyl ester was dissolved in 50 µL DMSO and added to 3.0 mL 0.1M NaHCO_3_ and mixed. A volume of 1.5 mL working solution was added to the moist 1.5 mL collagen beds and incubated overnight in the dark at 21°C. Plates were washed with PBS 4 mL 5× 30 minutes each and then washed with diH20 4 mL 5× 30 minutes each to remove unbound dye. A volume of 1.5 mL 0.06 N Acetic Acid was added to the plates which were then incubated overnight at 4°C to redissolve collagen. The solution was pipetted up and down to shear collagen and remove lumps. Collagen was pooled into 15 mL conical tube and mixed well with 3.0 mL 4°C C H2O. The solution was split into 1.5 mL micro centrifuge tubes and spun at ∼12K g for 30 minutes at 4°C and the pellet was discarded. This yielded ∼1.1 mg/mL collagen at a volume of 4.5 mL in 0.02N Acetic Acid.

Cells were passaged at approximately 50% confluency. To make 500 µL collagen gel, 50 µL 10×PBS pipetted and mixed in a microcentrifuge tube along with 7 µL 7.5% Sodium Bicarbonate, 5 µL PSF, 250 µL acid extracted rat tail tendon collagen I stock (BD Biosciences Discover Labware, Bedford, MA), and 83 µL fluorescent (CF633, Biotium) collagen and stored on ice. The appropriate volume of cell suspension corresponding to roughly 2,500 cells was added to the mixture followed by additional medium such that the volume was equal to 500 µL. On each plate, 100 µL of collagen-cell solution was carefully spread around the surface of the PAA and then allowed to polymerize in room temperature for 20 minutes. After that time, 2mL media was gently pipetted into the plate to avoid tearing the collagen gel.

To determine if the collagen gels were similar from experiment to experiment, the average mesh sizes of each collagen network was compared. The average mesh size for the GFP-wt-Src cells was 12.1 µm (standard deviation 1.3 µm) and for GFP-ca-Src cells was 11.7 µm (standard deviation 0.69 µm).

### Colocalization

Cells were prepared in collagen as described above and plated in two wells of a six well plate at a volume of 250 µL each. After ten minutes at room temperature, media was added and the cells were incubated for six hours at 37°C. After that time, the media was carefully aspirated and cells were fixed in 2 mL of a 3% paraformaldehyde/0.1% Triton-X 100 solution for 20 minutes at room temperature. Meanwhile, the vial contents of Alexa-Fluor 568 Phalloidin (Invitrogen, 300 units) were dissolved in 1.5 mL of methanol to create a stock solution. The fixing agent was then aspirated and a solution of 200 µL PBS and 10 µL stock phalloidin solution was added to each well. Cells were then stored at 4°C overnight. In the morning, cells were imaged with a 60x/1.4 N.A. Nikon water objective at 0.1 µm z-steps.

### Collagen Average Mesh Size Calculation

3D image stacks of collagen matrices were analyzed using a custom MATLAB (The MathWorks, Natick, MA) algorithm that performed the following analysis. First, a 3×3 median filter was applied to each slice. Then a binary intensity threshold was applied to all slices of a stack such that collagen fibrils were clearly discernible. Next, the distance between remaining fibril-containing pixels was counted in the x and y direction for each slice. According to Kaufman and colleagues [76], it is assumed the mesh size follows an exponential distribution exp(−*m*/*a*+*b*), where *m* is the mesh size and a the average mesh size. Thus the average mesh size *a* is the slope of the best-fit line to the semi-log histogram plot of mesh sizes. Because the largest and smallest possible measured mesh sizes are determined by the field of view and pixel noise respectively, the fit is applied across the ranges that looks most linear across all data sets, the ranges from 10 µm to 30 µm and 5 µm to 30 µm were both used, with no significant difference between them.

### Imaging

Between 12 and 24 hours after plating, the dishes were placed in a live cell chamber. Imaging was done using a spinning disk confocal microscope and captured with an Andor iXon digital camera using a 20× Nikon air objective (0.70 N.A.). The xy resolution was 512×512 pixels with pixel size 0.533 microns. Cells selected for imaging had nuclei oriented approximately 5 µm from the surface of the PAA gel and were also isolated from other cells by at least half the imaging plane (about 137 µm). A total of 21 GFP-wt-Src cells and 24 GFP-ca-Src cells were imaged and analyzed. Imaging lasted for 8 hours with three-channel capture every 20 minutes using a 1 µm z-step.

### FACS Staining

Cells were passaged at 50% confluency and centrifuged in 2 mL PBS to remove trypsin. The appropriate volume of solution containing 500,000 cells was resuspended in 100 µL PBS and placed on ice. A volume of 5 µL Phycoerythrin (PE)-conjugated mouse monoclonal anti-human MMP-14/MT1-MMP (R&D Systems, 100 tests) was added to the suspension and incubated for 30 minutes on ice. After that time, the cells were washed twice with 2 ml PBS so as to remove unbound antibody. After the final wash, the cells were resuspended in 500 µL PBS for immediate FACS analysis.

### Image Processing and Statistics

Image stacks were imported into Imaris (Bitplane AG, Zurich, Switzerland) for analysis. Drift correction, particle tracking, and surface tracking were done using autoregressive tracking algorithms. Particle tracks were imported to MATLAB where a custom traction force routine was implemented.

### Traction Force Theory and Application

For a linear, elastic, isotropic material for which body deformation is cause by external forces applied to its surface, the equations of equilibrium are given by.
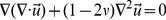
(1)where 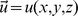 is the displacement vector and 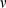 the Poisson ratio, taken here to be 0.45 for polyacrylamide gel [77]. A solution to this equation is sought by expressing the displacements by their Fourier transforms. Invoking the convolution theorem, this equation can be solved in Fourier space along with the three boundary conditions 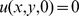 at the polyacrylamide-glass interface and the measured displacements of the tracer particles just below the interface at 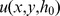, where for convenience we have assumed that the tracer particles lie in the same plane. A zero stress reference position for each bead is defined as the median of its location at all timepoints. The three-dimensional stresses are then related to the displacements through the constitutive equations in Fourier space by a tensor product,
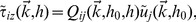
(2)where 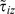 and 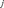 are the Fourier transforms of the component stress tensor and displacement vector respectively, and 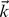 is the wave vector in the plane and the tensor 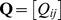 is calculated as in Xu et al. [78]. The inverse Fourier transform of 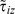 provides the traction forces at each point on the surface in the direction of i.

## Supporting Information

Figure S1 **Rheometry of the PAA with varying bead density.** We demonstrate that the embedded bead density has a minimal impact on the bulk polyacrylamide gel response to applied forces. In a cone-plate geometry, we apply a 1% oscillatory shear with 1 Hz frequency to polymerizing polyacrylamide gels with bead volumes ranging from 0–60 µL (Fig. 1), i.e., up to 4 times the reported amount in the submitted manuscript. Our results show that the plateau storage moduli fluctuate over a mean value ≅80.8 Pa (Fig. 2) with a standard error ≅ 3.8 Pa, or ≅ 4.7% of the mean value, on the order of sample-to-sample variations. We image the polymerized polyacrylamide gels *in situ* using a confocal-rheometer and observe a definitive increase in bead density throughout the sheared volume (Fig. 2, *insets*). Particle peak finding routines reveal a slightly more than four-fold increase in bead density when comparing the V = 5 µL and V = 60 µL gel cases. Our rheological results do not suggest any monotonic deviation from the average measured modulus across a wide range of bead densities.
(TIF)

Figure S2 **Gallery z-slice views of example fixed cells.** Examples of each cell line fixed for the Src localization analysis (A) eGFP-ca-Src (B) eGFP-wt-Src. **Cyan**: collagen fibrils. **Yellow**: Actin labled by Alexa-Fluor 568 Phalloidin. **Magenta**: eGFP-Src In both cell types, actin is localized primarily at cell periphery. In cells with eGFP-ca-Src (top) Src localized to the cell periphery, preferentially to membrane protrusions. In cells with eGFP-wt-Src (bottom) Src localized to the cell interior, preferentially to membrane protrusions. Use of a higher-N.A. objective in the live-cell allows for better resolution of both Src position (see methods).
(TIF)

Movie S1 **A z-stack of spinning disk images that was used to create** **Fig. 2** **A, a 3D reconstruction of a MDA-MB-231/GFP-ca-Src cell cultured in collagen for approximately 2 h.**
(AVI)

Movie S2 **A movie derived from** **Fig. 2** **B in which images were acquired every 10 sec during a period of 33 minutes in a live imaging experiment in which GFP-ca-Src cells (clone C1G1) were cultured on glass.**
(AVI)

Movie S3 **A movie derived from** **Fig. 2** **B in which images were acquired every 5 min during a period of 20 min in a live imaging experiment in which GFP-ca-Src cells (clone C2E8) were cultured on glass.**
(AVI)

Movie S4 **A movie from which** **Fig. 5** **was derived, showing the full sequence of the GFP-wt-Src cell.**
(AVI)

Movie S5 **A movie from which** **Fig. 5** **was derived, showing the full sequence of the GFP-ca-Src cell.**
(MOV)

Movie S6 **A movie from which** **Fig. 6** **was derived, showing the full sequence of the GFP-wt-Src cell.**
(AVI)

Movie S7 **A movie from which** **Fig. 6** **was derived, showing the full sequence of the GFP-ca-Src cell.**
(AVI)

Movie S8 **Rotating 3D view of the cell from** **Fig. 3** **. A.** **Cyan**: collagen fibrils. **Yellow**: Actin labled by Alexa-Fluor 568 Phalloidin. **Magenta**: eGFP-ca-Src. In both cell types, F-actin is localized primarily at the cell periphery. See Fig. S2 for gallery views of individual z-slices, and Movies S8 and S9 for rotating 3D views. Cells were fixed after 6 hours in the 3D collagen network. Use of a higher-N.A. objective in the live-cell allows for better resolution of both Src position (see methods). The actin channel is turned off briefly at the end to maximize visibility.
(AVI)

Movie S9 **Rotating 3D view of the cell from** **Fig. 3** **B.** **Cyan**: collagen fibrils. **Yellow**: Actin labled by Alexa-Fluor 568 Phalloidin. **Magenta**: eGFP-wt-Src. In both cell types, F-actin is localized primarily at the cell periphery. See Fig. S2 for gallery views of individual z-slices, and Movies S8 and S9 for rotating 3D views. Cells were fixed after 6 hours in the 3D collagen network. Use of a higher-N.A. objective in the live-cell allows for better resolution of both Src position (see methods). The actin channel is turned off briefly at the end to maximize visibility.
(AVI)

File S1 **Contains all Matlab files used in the 3D traction force calculations and a detailed protocol for creation of the 3D fibrillar collagen gel adhered to a polyacrylamide gel system in a MatTek 6-well glass-bottom dish.**
(ZIP)
