# Supplementary figures and images for: A Novel 3D Fibril Force Assay Implicates Src in Tumor Cell Force Generation in Collagen Networks
Source: PLoS One. 2013 Mar 11;8(3):e58138. doi: 10.1371/journal.pone.0058138 (PMC3594227; doi:10.1371/journal.pone.0058138)

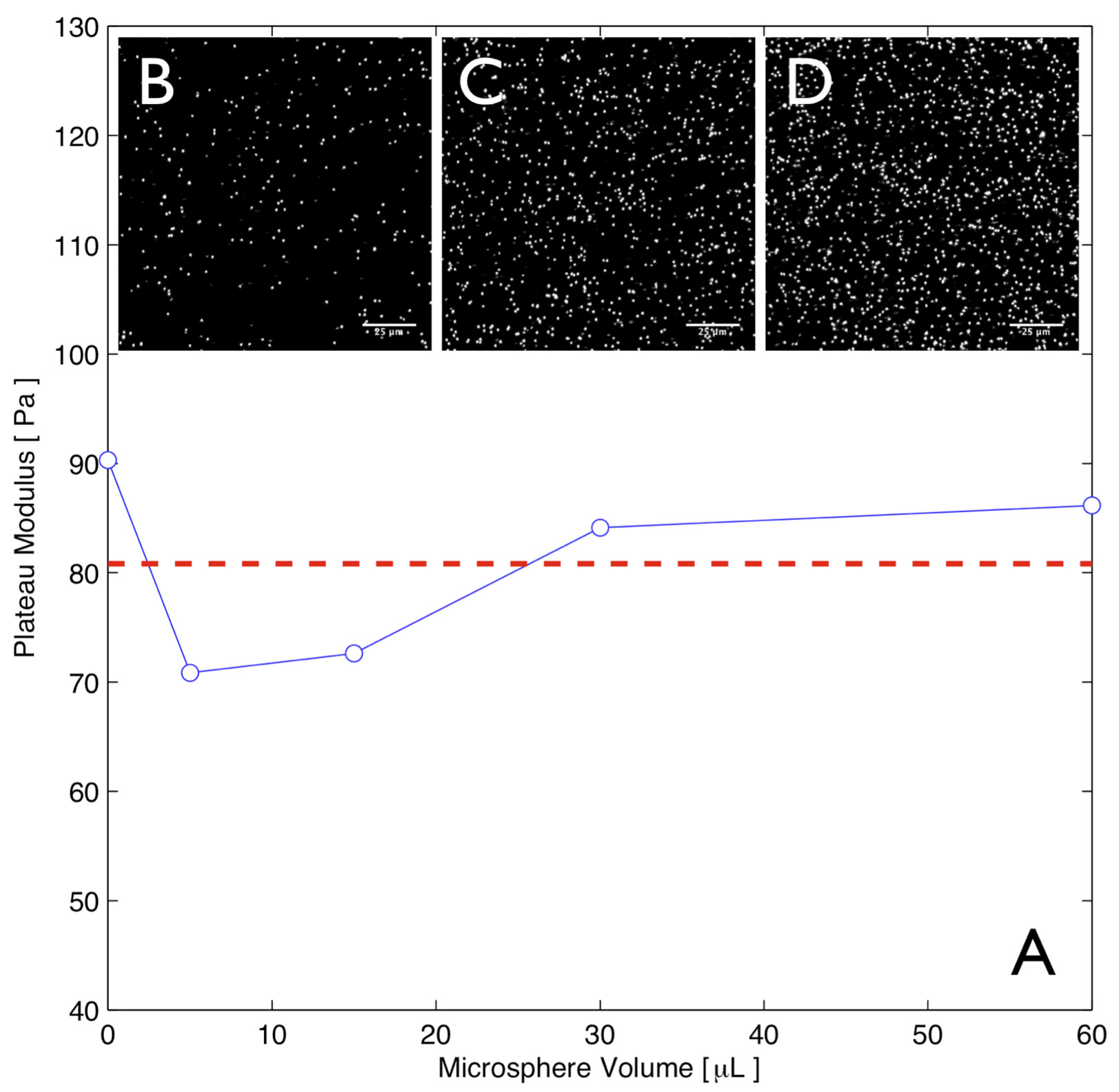

Supplement: Figure S1 — Rheometry of the PAA with varying bead density. We demonstrate that the embedded bead density has a minimal impact on the bulk polyacrylamide gel response to applied forces. In a cone-plate geometry, we apply a 1% oscillatory shear with 1 Hz frequency to polymerizing polyacrylamide gels with bead volumes ranging from 0–60 µL (Fig. 1), i.e., up to 4 times the reported amount in the submitted manuscript. Our results show that the plateau storage moduli fluctuate over a mean value ≅80.8 Pa (Fig. 2) with a standard error ≅ 3.8 Pa, or ≅ 4.7% of the mean value, on the order of sample-to-sample variations. We image the polymerized polyacrylamide gels in situ using a confocal-rheometer and observe a definitive increase in bead density throughout the sheared volume (Fig. 2, insets). Particle peak finding routines reveal a slightly more than four-fold increase in bead density when comparing the V = 5 µL and V = 60 µL gel cases. Our rheological results do not suggest any monotonic deviation from the average measured modulus across a wide range of bead densities. (TIF) [file pone.0058138.s001.tif]

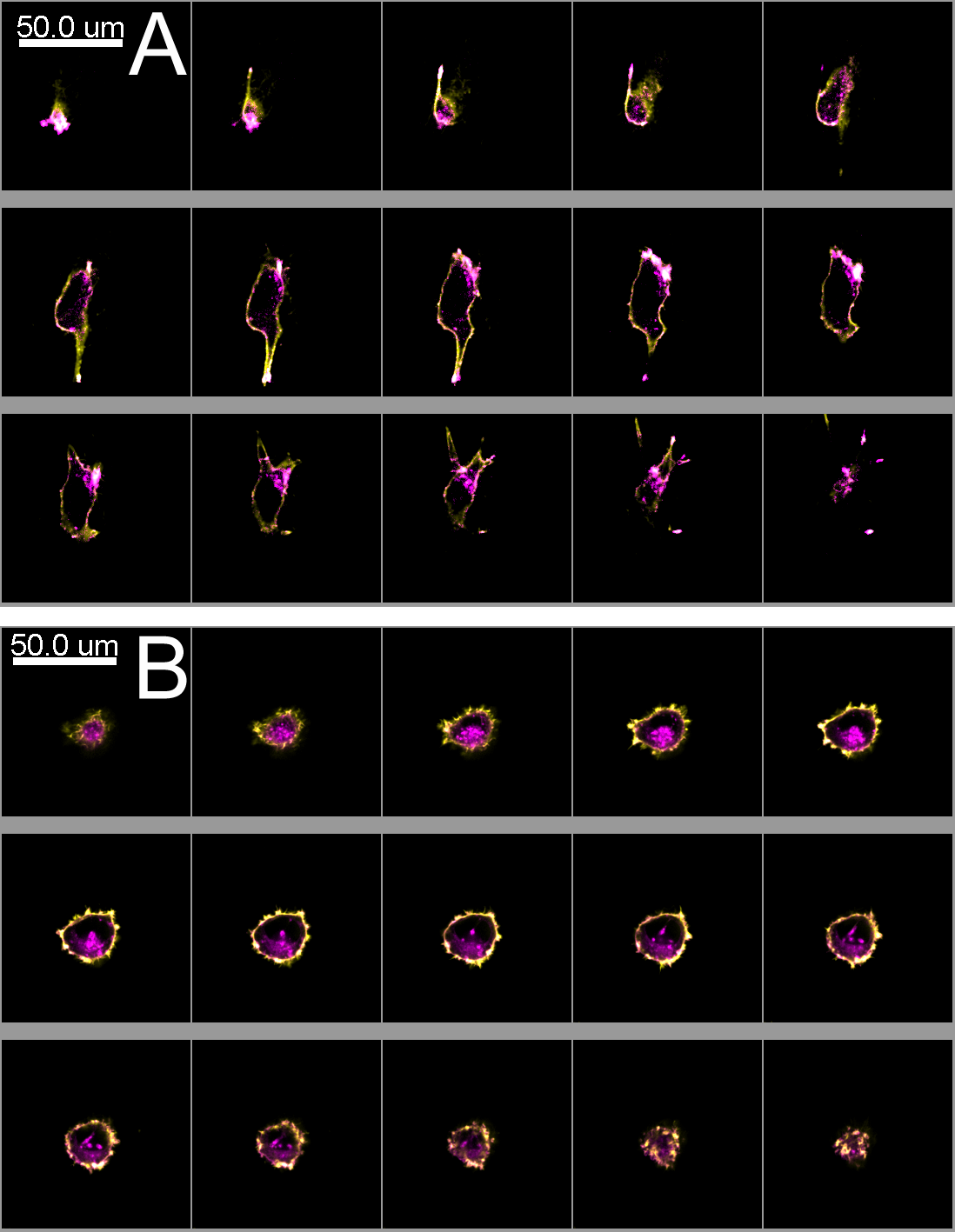

Supplement: Figure S2 — Gallery z-slice views of example fixed cells. Examples of each cell line fixed for the Src localization analysis (A) eGFP-ca-Src (B) eGFP-wt-Src. Cyan: collagen fibrils. Yellow: Actin labled by Alexa-Fluor 568 Phalloidin. Magenta: eGFP-Src In both cell types, actin is localized primarily at cell periphery. In cells with eGFP-ca-Src (top) Src localized to the cell periphery, preferentially to membrane protrusions. In cells with eGFP-wt-Src (bottom) Src localized to the cell interior, preferentially to membrane protrusions. Use of a higher-N.A. objective in the live-cell allows for better resolution of both Src position (see methods). (TIF) [file pone.0058138.s002.tif]
