# Supplementary material for: A Novel 3D Fibril Force Assay Implicates Src in Tumor Cell Force Generation in Collagen Networks
Source: PLoS One. 2013 Mar 11;8(3):e58138. doi: 10.1371/journal.pone.0058138 (PMC3594227; doi:10.1371/journal.pone.0058138)
Supplement: File S1 — Contains all Matlab files used in the 3D traction force calculations and a detailed protocol for creation of the 3D fibrillar collagen gel adhered to a polyacrylamide gel system in a MatTek 6-well glass-bottom dish. (ZIP) [file pone.0058138.s012.zip › Protocol and Matlab Files/3D Collagen on PAA Traction Force General Protocol.doc]

**Functionalize 6-well plates, top coverslips**

**Functionalize Bottom Coverslips w/ Cell Tak**

1. Under the hood, put Cell-Tak, 0.1M Sodium Bicarbonate, 1N NaOH on ice.
2. Pipette 3mL 0.1M Sodium Bicarb and 60 μl Cell-Tak into centrifuge tube and vortex gently.
3. Pipette 30 μl 1N NaOH into working solution and vortex gently again.
4. Within ten minutes of solution prep, pipette 500 μl working solution onto each bottom coverslip of 6-well plate and spread gently using pipette tip such that solution covers entire coverslip area.
5. Allow 20 minutes for Cell-Tak solution to functionalize bottom coverslips.
6. After 20 minutes, wash bottom coverslips 3 times each with DiH2O and allow to air dry under hood.

**Make Top Coverslips Hydrophobic**

1. Wipe down six 18mm glass coverslips using an ethanol-sprayed piece of Kim-Wipe between tweezers. Allow to air dry.
2. Submerge coverslips in Sigmacote for 3 minutes.
3. Place coverslips in a coverslip rack and wash in a beaker of diH2O for ten minutes. Pipette the remaining Sigmacote back into the bottle if it is clean as it can be used again.
4. Allow the top coverslips to air dry and dispose of wash water into the appropriate chemical waste bin.

**~100 PA Polyacrylamide (PAA) Preparation/Plating**

1. Put 40% Bio-Rad Acrylamyde, 1% BIS-Acrylamyde, 1x PBS, 5μm diameter microbead solution on ice.
2. Pipette 840 μl PBS, 75 μl 40% Acrylamyde, 70μl BIS-Acrylamyde into microcentrifuge tube, put on ice.
3. Sonicate bead solution for 10 seconds on high power, then pipette 15 μl bead solution into working microcentrifuge tube solution.
4. Mix working microcentrifuge tube solution gently by inverting, then dessicate solution for 15 minutes.
5. After dessication, store on ice for five minutes.
6. Pipette 3 μl TEMED followed by 10 μl 10% Ammonium Persulfate into microcentrifuge solution. This initiates polymerization. Put solution back on ice and place under hood along with dry,functionalized top coverslips.
7. Under the hood, pipette 5.5 μl PAA solution onto the middle of one of the bottom coverslips and immediately but carefully use tweezers to place functionalized top coverslip on top of the PAA gel. If necessary, very gently use tweezers to apply pressure to top coverslip so that the PAA gel spreads evenly under the area of the top coverslip. Repeat this process for the rest of the wells within five minutes.
8. After each top coverslip has been applied, turn the six-well plate upside down and cover with a wet paper towel. This provides a humid environment for the gels to polymerize in. Allow gels to polymerize in this way for 15-20 minutes.
9. After gels have polymerized, pipette 3ml DiH2O into each well and let sit under hood for about 20 minutes.
10. To remove top coverslips, take tweezers and very gently push each coverslip horizontally back and forth along the surface of the PAA gel until it feels possible to use the tweezers to pull the coverslip off the surface of the gel without ripping the gel. To store gels overnight, add 30 μl PSF to the 3 ml DiH2O in each well.

Functionalize PAA w/ Cell-Tak

To functionalize PAA for collagen plating, follow the protocol above to prepare Cell-Tak solution. Before plating solution onto PAA gels, make sure there is no water left on the gel surface. It may be necessary to remove the water from the well and then wait one minute for only the surface of the PAA to dry slightly. If there is still water residue on the bottom coverslip/on top of the PAA gel, it is possible that the Cell-Tak solution will not work. Again, apply 500 μl Cell-Tak solution to each well such that it covers both PAA gels and the entire area of the bottom coverslip and wait 20 minutes before washing three times with DiH2O as before. After washing, reapply DiH2O to each well so that the PAA gels do not dry out.

Mix 500μl collagen/cell stock; Plate:

1. Put 10x PBS, PSF, diH2O, 7.5% sodium bicarbonate, collagen stock and fluorescent collagen on ice under hood.
2. For 500 μl cell/collagen mix: pipette 50 μl 10x PBS, 7μl sodium bicarb, 5μl PSF, 250μl collagen stock, 83μl fluorescent collagen into a microcentrifuge tube; leave on ice.
3. Cells need to be plated at a concentration of 5000 cells per plate. Pipette 20 μl cell suspension onto hemocytometer and take the average of the mm x mm square counts. 25,000/(avg count # x 10^4) is the volume (ml) of cell suspension to add to the working collagen mix. After adding the cell suspension volume, pipette the appropriate volume of media into the working mix such that the total volume is 500 μl.
4. Gently invert the microcentrifuge tube a few times to mix the cells throughout the collagen solution. Place the micro centrifuge tube back on ice. Do not leave the solution off ice for more than ten seconds as the collagen begins polymerization as soon as the solution is above freezing. The solution is now ready for plating.
5. Totally remove the diH20 from a well. If possible, vacuum excess water off the surface of the PAA gel without tearing the gel, as a small layer of water will impede the binding of the collagen to the PAA surface. While the gel is still exposed, quickly pipette 100 μl collagen/cell solution onto the surface of the PAA gel. It is important to do this quickly as the PAA gel immediately beings to dehydrate and can totally evaporate if left exposed for longer than a minute. Use your pipette tip to quickly but gently spread the mix such that it covers the entire area of the bottom glass coverslip, including the PAA gel. Note the time of plating. Repeat for the remaining four wells. Note the time of plating for the last well.
6. Allow collagen mix in each well to polymerize between 15-20 minutes in room temperature. Polymerization periods longer than 20 minutes will result in dehydrated gels which can easily crack or tear. The polymerization times for each well should be the same. Beginning with the first plated well, very slowly pipette about 100 μl media onto the center of the collagen mix. The collagen gel is extremely fragile and extra care must be taken to ensure it is not ripped or broken by the application of the media. The preliminary application of a small volume of media hydrates the gel and helps to prevent tearing as well as allows one the time to hydrate all wells in the necessary timeframe.
7. Carefully place the wells in the live cell chamber on the microscope stage. Gently pipette enough media so that the bottom of the well is totally submerged (1-2ml). Incubate on the stage for at least four hours before imaging.
